# Branding a New Technological Outlook for Future Orthopaedics

**DOI:** 10.3390/bioengineering12050494

**Published:** 2025-05-07

**Authors:** Nicole Tueni, Farid Amirouche

**Affiliations:** 1 Institute of Continuum Mechanics and Biomechanics, Friedrich-Alexander-Universität Erlangen-Nuremberg, 91054 Erlangen, Germany; nicole.tueni@fau.de; 2 Department of Orthopaedics, University of Illinois at Chicago, Chicago, IL 60612, USA; 3 Department of Orthopaedic Surgery, Northshore University HealthSystem, Skokie, IL 60076, USA

**Keywords:** orthopedic innovation, technological innovation, personalized medicine, robotic surgery, 3D printing in orthopedics, artificial intelligence in healthcare, virtual surgical planning

## Abstract

Orthopedics is undergoing a transformative shift driven by personalized medical technologies that enhance precision, efficiency, and patient outcomes. Virtual surgical planning, robotic assistance, and real-time 3D navigation have revolutionized procedures like total knee arthroplasty and hip replacement, offering unparalleled accuracy and reducing recovery times. Integrating artificial intelligence, advanced imaging, and 3D-printed patient-specific implants further elevates surgical precision, minimizes intraoperative complications, and supports individualized care. In sports orthopedics, wearable sensors and motion analysis technologies are revolutionizing diagnostics, injury prevention, and rehabilitation, enabling real-time decision-making and improved patient safety. Health-tracking devices are advancing recovery and supporting preventative care, transforming athletic performance management. Concurrently, breakthroughs in biologics, biomaterials, and bioprinting are reshaping treatments for cartilage defects, ligament injuries, osteoporosis, and meniscal damage. These innovations are poised to establish new benchmarks for regenerative medicine in orthopedics. By combining cutting-edge technologies with interdisciplinary collaboration, the field is redefining surgical standards, optimizing patient care, and paving the way for a highly personalized and efficient future.

## 1. Introduction

Orthopedic research is a collaborative endeavor that bridges two key domains: clinical expertise and technological innovation. Clinical practitioners, including physical therapists, doctors, and pharmacists, offer valuable insights from their patient interactions, identifying recurring conditions and emerging challenges. This practical knowledge drives innovation by highlighting areas for further exploration and improvement [1,2].

At the same time, advancements in medical engineering, biomaterials, and biomechanics provide new tools to address these challenges. From nanoscale biosensors to multidisciplinary molecular research, technical specialists collaborate closely with clinicians to develop and deliver tailored solutions, enhancing patient care and effectively managing costs [3,4]. This paper examines how such collaborations shape orthopedics’ future, particularly in imaging, surgical planning, and rehabilitation.

In an era where patients are increasingly informed and proactive about their healthcare options, staying abreast of research is vital [5,6]. This knowledge enables hospitals and clinics to adapt to evolving demands, including integrating wireless and intelligent technologies. Recent developments in telemedicine and virtual hospitals are promising innovations that could soon serve as essential platforms for outreach communities while complementing specialty care in modern healthcare systems [7,8] (World Health Organization, 2021). Furthermore, advancements in imaging technologies, such as 3D visualization and augmented reality for surgical planning, are paving the way for precision medicine in orthopedics [9,10].

The mission of orthopedics is deeply rooted in patient care: to provide the finest care possible by addressing pain, enhancing mobility, and ensuring safety, comfort, accessibility, convenience, and affordability [11]. Through the collaboration of clinical and technical expertise, the field is meeting the needs of today’s patients and paving the way for future innovations that will redefine healthcare delivery.

Building upon this foundational premise, this paper explores the dynamic evolution of orthopedic practices, highlighting the profound impact of recent advancements in imaging technologies, the strategic use of virtual planning, and groundbreaking surgical innovations [12]. These trends are reshaping the landscape of orthopedics and enhancing patient outcomes and operational efficiencies within the field.

## 2. Trends in Orthopedic Research

Orthopedic research focuses on understanding the human musculoskeletal system, which is defined as an interconnected network of bones, muscles, tendons, cartilage, and nerves that support body mechanics, maintain posture and stability, and provide necessary movement.

Clinical orthopedics offers solutions to musculoskeletal injuries across various specialties, including sports medicine, rehabilitation, spine care, deformity correction, pediatrics, and hand care.

Over the past decade, advancements have been made to meet the demands of modern surgery, facilitating procedures with fewer complications and greater precision [13].

**Figure 1:**
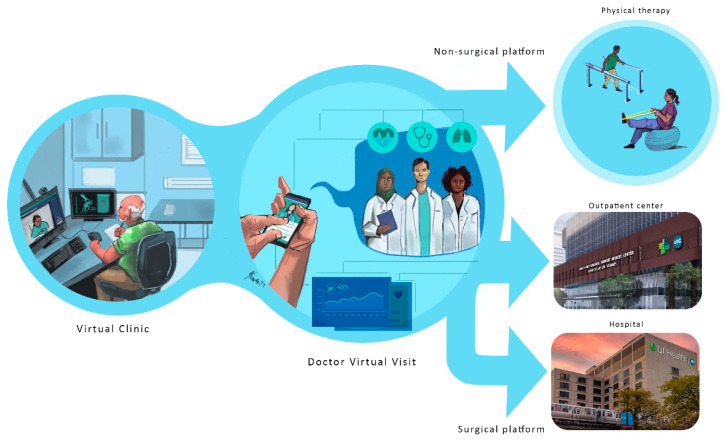
Virtual clinic workflow: Initial imaging and tests at outpatient clinics are sent to physicians for virtual consultation, expediting diagnosis and reducing hospital wait times.

Now, orthopedic surgery is shifting towards less invasive and more precise procedures, demanding innovative tools with sensory feedback and integrating advanced imaging with visual navigation systems [14]. This intelligent platform design is constantly evolving and is bolstered by modern technologies, rendering a more fluid and accelerated process. Figure 1 demonstrates this virtual clinic interaction. Initial imaging is performed at outpatient clinics or pharmacies, where medical history and additional tests are conducted to complete the assessment. The medical file is then virtually sent to the physician for consultation and diagnosis, significantly reducing hospital waiting times.

The process is divided into six phases:

- Diagnosis phase;
- Planning phase;
- Validation phase;
- Surgical phase;
- Post-surgical phase;
- Orthopedic platform and patient feedback.

Each phase is discussed in detail below.

### 2.1. Diagnosis Phase

Diagnosis involves analyzing patient-specific data, such as symptoms and imaging results, to determine the underlying cause of a condition. Advanced imaging tools augmented by digital twins, patient-reported outcome measures, and wearable devices further enhance this phase.

#### 2.1.1. Imaging and 3D Tools

In the diagnosis phase, imaging techniques such as X-ray Computed Tomography (CT) scans or Magnetic Resonance Imaging (MRI) scans are combined with analysis tools, including automated morphometric measurement, image segmentation, and landmark detection. Currently, researchers are constructing patient-specific models, also known as digital twins [15,16]. These are high-fidelity models of organs and systems created using patient-specific information. In addition to precisely reflecting the patient’s anatomy and condition, they may simulate alternative treatments and measure the appropriate tissue, bone, or organ reaction, such as tissue stresses or remodeling [17].

The relationship between diagnostic imaging techniques, such as Computed Tomography (CT), and outcomes in orthopedic treatments is profound, primarily due to their role in assessing tissue stress and strain and tissue remodeling [18].

- Understanding Tissue Stress and Remodeling:

Diagnostic imaging enables the detailed visualization of the structural integrity of bones, cartilage, and surrounding soft tissues. CT scans, in particular, provide high-resolution images that allow clinicians to evaluate the extent of injuries, disease progression, and the biomechanical environment accurately. By identifying areas of abnormal stress concentration, physicians can better understand how these stresses contribute to tissue remodeling.

- Tailored Treatment Planning:

Integrating imaging data with biomechanical analysis enables the creation of individualized treatment plans. For example, suppose a CT scan reveals excessive stress on a particular joint due to malalignment or degenerative changes. In that case, a surgeon may opt for corrective procedures or specific rehabilitation protocols designed to redistribute forces more evenly across the joint. This tailored approach can improve surgical outcomes and enhance recovery.

- Monitoring Healing and Rehabilitation:

Advanced imaging not only aids in diagnosis but also plays a crucial role in monitoring patient progress after surgery. Using follow-up CT scans, clinicians can observe how tissues are remodeling in response to treatment. Suppose imaging shows that the healing process is not progressing as expected or that there are signs of stress-related complications. In that case, timely interventions can be implemented to adjust rehabilitation strategies or reconsider surgical options, potentially improving outcomes.

- Predicting Complications:

Visualizing changes in tissue composition and structure over time enables clinicians to predict potential complications, such as non-unions in fracture healing or the long-term impact of overuse injuries. For instance, CT imaging can reveal early signs of stress fractures or cartilage degradation, prompting early interventions that may prevent more severe injuries and lead to better long-term functionality.

- Enhancing Decision-Making with Digital Twins:

The concept of digital twins—patient-specific models based on imaging studies—offers even more nuanced insights. These models simulate how tissues may respond to various surgical interventions or rehabilitation protocols, allowing for scenario testing. This technology provides visual validation for specific treatment paths and enhances the understanding of how adjustments in the biomechanical environment can lead to favorable outcomes.

- Research and Development of New Treatments

A comprehensive understanding of the relationship between diagnostic imaging, tissue stress, and remodeling paves the way for developing innovative orthopedic treatments. For example, researchers can investigate how various surgical techniques or rehabilitation approaches affect tissue adaptation, continually utilizing imaging as a feedback mechanism to refine their practices.

In conclusion, the nexus between diagnostic imaging, tissue stress, and remodeling is critical in optimizing orthopedic treatments. By leveraging advanced imaging techniques, clinicians can significantly enhance diagnostic accuracy, tailor interventions, monitor recovery, and ultimately improve patient outcomes in orthopedic care.

During this phase, more advanced technologies are utilized to provide insight into the patient’s condition and to provide the AI system with additional patient data. Fluoroscopic procedures produce continuous images of a patient’s musculoskeletal system, allowing for monitoring movement over time [19]. Electromyography is used to diagnose muscular problems by recording the electrical activity of muscle tissue as a visual display or an aural signal using electrodes placed on the skin or muscle [20]. As suggested by its name, absorptiometry measures the quantity of radiation absorbed by bone to calculate its density [21]. Ultrasonography detects vascular problems with high-energy sound waves [22,23]. Through a small incision, photos and videos of a damaged area are captured during arthroscopy.

#### 2.1.2. Surveys, Sensors, and Smartphones

As new equipment and techniques enter orthopedic practice, accurate and comparable methods of assessing the quality of care and results will be paramount. The future success of orthopedic surgery will be evaluated using patient-reported outcome measures with digital enhancements (PROMs) [24]. Patients can be offered more pertinent questions with fewer responses by employing Item Response Theory and Computer Adaptive Testing (CAT) [25,26], which tailors the remaining survey questions depending on those already answered. CAT can make patient survey replies more effective, of better quality, and easily comparable. Additionally, MIS CAT assessments can help avoid the ceiling effects observed in conventional musculoskeletal function ratings. As existing models for measuring pre-intervention function, treatment progress, and outcomes are enhanced, cell phones, wearables, and sensors are contributing additional layers of objective, real-world data, allowing for more in-depth and nuanced knowledge of musculoskeletal treatments and outcomes [27,28]. Increasingly, specialized sensors that offer continuous, real-time data will improve decision-making before, during, and after surgery.

Smartphones are rapidly becoming ubiquitous worldwide, and their built-in tracking of steps and activity provides a convenient method for assessing individuals’ physical activity. Patient-specific data obtained will shed new light on recovery trends, improving pre-surgical planning and post-surgical monitoring [29,30,31].

#### 2.1.3. Picture Archiving and Communications Systems

Picture Archiving and Communications Systems (PACSs) [32] are electronic systems that store and organize patient-related photos, data, and reports. They provide a chronological view of a patient’s medical history, thus enhancing the quality of care and accelerating the workflow.

Such systems are essential for remote diagnosis because they allow clinicians stationed in distant regions to access patients’ information.

Once a precise diagnosis is established, the planning phase utilizes advanced tools, such as augmented reality and artificial intelligence, to customize preoperative strategies and ensure optimized outcomes tailored to the patient’s condition.

### 2.2. Planning Phase

Preoperative evaluation is a crucial phase that, if completed correctly, can enhance the patient’s experience and quality of care by allowing the medical staff to evaluate and prepare each treatment step, thereby decreasing the chance of complications [33].

Historically, pre-planning consisted of handwriting thorough “blueprints,” including symptoms and past medical, surgical, and family histories, and selecting the optimal treatment option and instruments. Currently, the advancement of engineering technology in orthopedics enables the application of innovative planning techniques that significantly influence decision-making [34,35], considering the patient’s medical state and the desired outcomes. What follows is a discussion of some of these technologies and their involvement in each step of the pre-planning phase.

Treatment planning is a complex optimization process that involves considering the diagnosis and producing a treatment plan to achieve the intended outcome.

#### 2.2.1. AR and Planning

The application of augmented reality technologies in medical training, preoperative planning, data visualization, and tool guidance is imminent. In the past few years, augmented reality technologies have transitioned from research laboratories to ordinary medical practice [36,37,38,39,40]. Engineered volumetric imaging techniques, such as CT scanning and MRI, are integrated with image analysis and processing software applications that reconstruct the images, producing a patient-specific 3D model of the organs. Furthermore, kinematic measurements from sensors and implantable devices generate real-time data about the orthopedic system components and their interactions (movement, force, flexibility, etc.), thereby revealing possible abnormalities. This combination of technologies gives clinicians a comprehensive view of the patient’s condition.

#### 2.2.2. Digital Templates

Digital templates comprise up-to-date databases of implants and biological materials. In conjunction with AI technology and designed instruments, the templates assist surgeons in calculating the precise sizes and materials of implants used during surgery. This saves time and cost by decreasing implant-related errors and waste caused by improper dimensioning [41,42].

The operation logistics phase concludes the planning process. It involves preparing the surgical site, instruments, and implants, among other things, to ensure the procedure proceeds smoothly and efficiently. As with the preceding steps, this process has undergone significant changes due to the introduction of new methodologies and technologies.

#### 2.2.3. AI and Logistics

Most fields use AI-based solutions to automate and accelerate time-consuming procedures. Specifically, in orthopedics, AI systems utilize vast amounts of data that enable medical teams to explore numerous scenarios, forecast surgery outcomes, and select the most appropriate treatment, thereby providing patients with the highest level of individualized care [43,44].

Additionally, AI is making progress in creating customized medical devices. In recent years, personalized implants and guides created from patient-specific scan data have increased, particularly for surgeries such as spinal fusion [45,46]. Additionally, Johnson & Johnson and others are deploying 3D-printed patient-specific surgical guides, which offer numerous benefits in orthopedics.

Moreover, AI-based systems and digital templates are used to select the appropriate instrumentation and other surgical-related tools, thereby reducing surgery cost by eliminating hospital overstocking, which leads to waste due to product expiration, and optimizing the number of instruments to sterilize.

AI-based systems and digital templates are crucial in modern surgery and treatment planning, particularly in orthopedics.

AI-based systems leverage large datasets and complex algorithms to enhance decision-making and improve outcomes in medical procedures. These systems analyze historical and real-time data to provide insights into various surgical scenarios, predict potential outcomes, and help medical teams choose the most effective treatment options. In orthopedics, AI applications have been pivotal in personalizing care, as they can assess a patient’s unique anatomy and medical history. For instance, AI can assist in identifying surgical risks and suggesting tailored intervention strategies, ultimately improving patient care and optimizing surgical processes.

Digital templates consist of comprehensive, up-to-date databases with information on implants and biological materials. By integrating these templates with AI technology, surgeons can determine the precise sizes and specifications of implants needed for a particular procedure. This technology streamlines the planning process, reducing the risk of errors associated with implant dimensioning. By ensuring the accurate selection of materials, digital templates help minimize waste and costs related to incorrect implant sizes and enhance the efficiency of surgical procedures. Together, AI-based systems and digital templates improve the accuracy of surgical planning and contribute to better patient outcomes and operational effectiveness in healthcare settings.

#### 2.2.4. Ambulatory Surgery Centers

Ambulatory surgery centers (ASCs) are becoming increasingly integral to orthopedic surgical plans, as several surgeries are now performed in outpatient settings. The main advantage is that they do not require hospital admission; hence, they focus on offering economical services in a less stressful setting than what many regular hospitals can offer [47].

**Figure 2:**
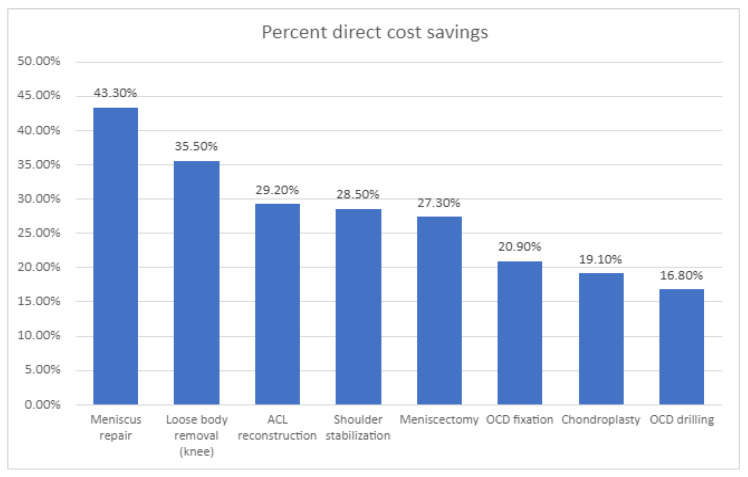
The percentages of direct cost savings for eight specific surgical procedures conducted at an ambulatory surgery center compared to those performed at a university-based children’s hospital highlight significant differences in surgical expenses. This analysis includes procedures such as anterior cruciate ligament (ACL) repair and the treatment of osteochondritis dissecans (OCD), as noted in the study by Fabricant et al. (2016). The findings provide valuable insights into the financial advantages of utilizing ambulatory surgery centers for pediatric surgical interventions.

Due to their limited storage space, restricted clean-room capabilities, and smaller operating rooms, ASCs are often more cost-effective than traditional hospitals [48]. Medical teams must prioritize cost-effectiveness, reduce equipment stockpiles, and implement efficient processes. An analysis of 1021 procedures in the Children’s Hospital of Philadelphia was performed [49], showing that orthopedic procedures had significantly lower direct costs when performed at the ASC, ranging from 17% to 43% of the price at the UH, as demonstrated in Figure 2. It was found that 80% of the cost savings were attributable to time. In comparison, 27% were attributable to supply utilization, and 73% of the operating-room time savings were attributed to surgical factors, while 27% were attributable to anesthesia factors.

After the surgical plan is developed, the validation phase is crucial in refining and confirming the protocol. It leverages virtual simulations and expert reviews to ensure procedural accuracy and minimize risks.

### 2.3. Validation Phase

**Figure 3:**
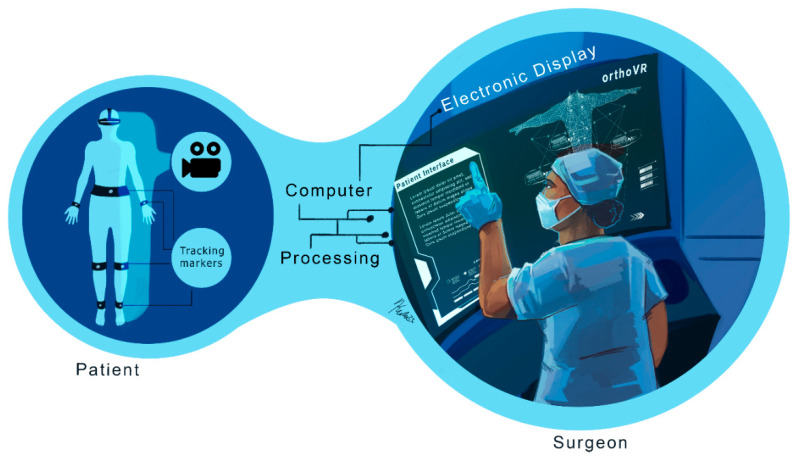
Schematic of augmented reality principles: Augmented reality (AR) and digital templates are crucial in enhancing virtual surgical protocol reviews. These technologies provide a detailed and immersive view of patient conditions, outlining critical aspects such as anatomical features, treatment steps, and comprehensive recovery plans. By integrating real-time data and 3D visualizations, AR enables medical professionals to execute treatments and surgical procedures with increased precision and confidence, ultimately improving patient outcomes and streamlining the surgical workflow.

The virtual visualization phase involves submitting the surgical protocol to field specialists for review, improvement, and approval. Augmented reality and digital templates can enhance this phase by providing teams with a comprehensive view of the patient’s condition, detailed treatment steps, and a recovery plan. This capability is highlighted in Figure 3, which provides a schematic diagram of AR-assisted planning. With surgical protocols validated virtually, the next step is to precisely execute these procedures using cutting-edge technologies in both the surgical and treatment phases.

Once the surgical protocol has been validated, the focus shifts to execution during the surgical phase, where cutting-edge technologies, such as robotics, augmented reality, and computer-assisted systems, enhance precision and reduce complications.

### 2.4. Surgical Phase

Orthopedic surgery focuses on musculoskeletal tissues. The surgery must be performed accurately to improve efficacy and reduce the risk of complications. Consequently, many mechanical instruments have been developed for use in various surgical procedures. However, since these tools are not specific to the morphology of each patient, they have limited precision.

Moreover, the complexity of the anatomy makes it difficult for surgeons to visualize the surgical site in 3D, complicating the surgery.

Computer-assisted surgery (CAS) has evolved into a valuable field that utilizes computer-enabled tracking systems and robotic devices to enhance reliability, reproducibility, and control during surgical procedures [50]. For example, images of the surgical site displayed on monitors in the operating room (OR) and vital signs enable the surgeon to receive real-time feedback on the surgical actions performed [51,52]. The integration of robotic systems enables greater precision during surgeries.

#### 2.4.1. Augmented Reality (AR) in CAS

**Figure 4:**
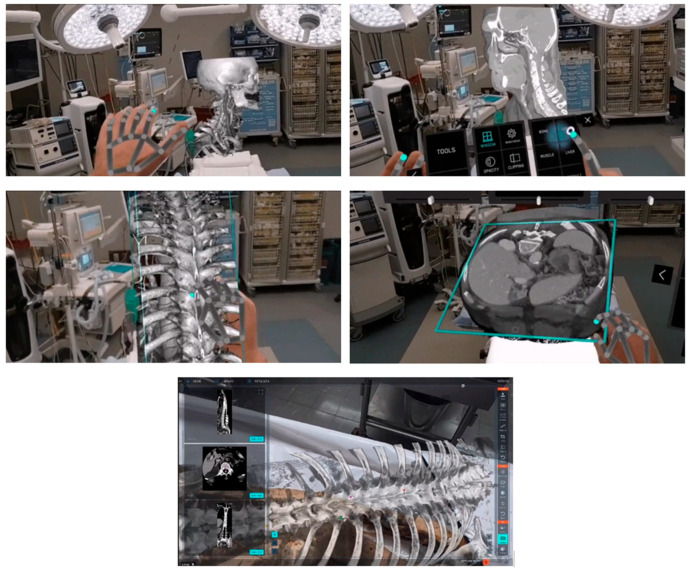
Virtual and augmented reality in healthcare is revolutionizing surgical procedures by providing advanced intraoperative scans that create detailed and high-resolution 3D models. These sophisticated models come equipped with automatic spine support, facilitating precise planning and navigation throughout the surgical process. High-resolution cameras play a crucial role by accurately detecting patient markers, ensuring the surgical team has clear and real-time identification of key anatomical features. Additionally, integrating 3D cone-beam Computed Tomography (CT) with live video images enhances the surgeon’s view, allowing for more effective decision-making and improved patient outcomes during complex operations.

In addition to being used in the pre-planning process, AR systems constitute an essential novel intraoperative data visualization tool for guidance. The surgeon is provided with a direct spatial interaction between medical images and the patient using augmented reality. The surgeon may view the patient and the photographs using augmented reality technology [53,54,55]. The images are superimposed on the patient, appearing in the same orientation and position as the corresponding anatomical features. Examining images in the exact position and orientation overlay on the patient enables the surgeon to visualize interior structures completely and thoroughly comprehend the patient’s unique anatomy and condition [56,57,58]. The authors of [59] created an accurate stereo camera-based augmented reality navigation system, which they then examined. The experiments demonstrated its precision and dependability. Philips recently introduced a new augmented reality solution, ClarifEye, that combines imaging and AR navigation. It utilizes an intraoperative scan to generate a high-resolution 3D model, providing proper support for accurate device selection, planning, and navigation by automatic spine segmentation. Additionally, four high-resolution cameras automatically detect non-invasive patient markers to enhance the surgical field, and a 3D cone-beam CT is superimposed on live video images. This advancement is clearly illustrated in Figure 4, which provides a detailed view of AR usage during surgery.

Modern technology, such as wearable Smart Glasses, is revolutionizing orthopedic surgery by enhancing AR and simplifying the lives of surgeons in the operating room. This advancement enables greater precision and reduces challenges during complex surgeries, as demonstrated by [60]. Our firm belief is that AR is not just a tool but the future of orthopedic surgery, promising exciting developments and advancements.

Some companies are actively working on integrating AR techniques into their platforms and utilizing the user-friendly, Google-developed ARCore. This system integrates virtual content with the actual environment, as viewed through a phone’s camera, offering significant capabilities: (i) motion tracking enables the phone to comprehend and monitor the rest of the environment; (ii) the phone’s environmental knowledge enables it to detect the size and placement of all types of surfaces; and (iii) light estimation allows the phone to estimate the current lighting conditions to see through AR masks with image registration video tracks that allow the display to superimpose an image on the exact treated location of the inpatient vs. heads-up display (a visualization method where data is displayed on the screen.

Furthermore, due to the numerous restrictions imposed by the COVID-19 emergency to prevent overcrowding and the presence of external personnel, it is becoming increasingly challenging to physically have technical and medical specialists present in the operating room, which is necessary for the regular accessibility of this information. This problem could be solved through the use of augmented reality surgery. Indeed, it can be efficiently utilized in various settings, enabling surgeons to remotely view the operating room directly through their own eyes and virtually perform or supervise an operation [61].

#### 2.4.2. Robotics in CAS

Robotic surgery enables surgeons to perform complex procedures with greater precision, flexibility, and control than traditional methods.

Multiple components comprise robotically assisted surgery (RAS) devices, including a console that enables surgeons to view a 3D reconstructed model of the surgical field and control the movement of surgical instruments [12,62,63]. This bedside system includes mechanical arms that hold and manipulate surgical tools as needed by the surgeon, along with additional carts that house and support the necessary hardware and software components.

**Figure 5:**
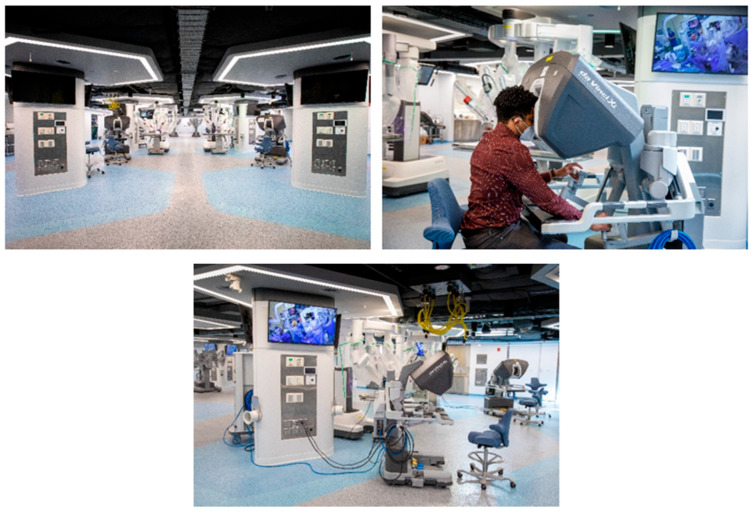
Surgical solutions utilizing multi-arm robotic technologies are revolutionizing medicine. (Source: Surgical Innovation Training Laboratory, University of Illinois at Chicago). These advanced robotic systems significantly enhance the precision of surgical procedures through their sophisticated visual feedback mechanisms and meticulously controlled movements, allowing surgeons to perform complex tasks with unparalleled accuracy and greater confidence.

During robot-assisted surgeries, the surgeon is seated near the patient. A tiny camera and surgical instruments are inserted through small incisions, allowing the surgeon to visualize the operative field on a video monitor and manually manipulate robotic arms that mimic the surgeon’s actions. These systems feature advanced visual feedback and controlled movements, as depicted in Figure 5, showcasing multi-arm robotic technologies. Robotic surgeries offer several advantages over conventional open surgery, including reduced pain, blood loss, infection risks, and decreased scarring [64,65]. Moreover, they offer shorter hospital stays and recovery times.

In addition to the benefits of image segmentation and developing 3D patient-specific models pre- and intraoperatively, image segmentation for 3D printing has become a key aspect of orthopedic surgery. Recently, with the advancement of artificial intelligence (AI) technology, organ anomalies can be accurately recognized and contoured from CT images or MRIs, and 3D objects can be rendered with precision [66,67].

Three-dimensional patient-specific constructs can be printed using rendered volumes, with the objective of using the patient’s cellular components to replace damaged or diseased tissue [16,68]. Three-dimensional bioprinting or regenerative medicine enables the local treatment of the region where the abnormality is found in a minimally invasive surgery (MIS) context, thereby avoiding complications and severe immune rejection.

The challenges in bioprinting remain in the ability to print cells that can survive, multiply, and build a functioning matrix on a tissue-specific scaffold that offers full support for the cells using growth factors that enhance cell development [69]. Cellink, a company under the BICO Group in GO, Sweden, is at the forefront of addressing these challenges. Their products frequently integrate skills in artificial intelligence, robotics, and diagnostics, with a focus on commercializing technology for life science research and bioprinting [70]. Similarly, Cellbricks, a company based in Berlin, Germany, in collaboration with the Berlin Institute of Health at Charité (BIH), is pioneering the replication of human tissues such as cartilage and bones by combining Synthetic Biology and 3D bioprinting.

In addition to bioprinting, collaborations between physicians and scientists have led to the discovery of non-invasive treatments, such as stem cell therapy, which consists of guiding stem cells placed in patients to replace and repair diseased cells and create healthy cells over time [71,72,73], and exosomes, which contain valuable molecular components of the cell of origin, such as proteins and RNA [74,75,76]. Exosome treatment can be administered intravenously, hence enhancing cell communication.

The transition from surgery to recovery is marked by the post-surgical phase, where advancements in rehabilitation tools, wearable technologies, and virtual reality ensure effective healing and faster restoration of mobility.

### 2.5. Post-Surgical Phase

The postoperative phase of a surgical procedure begins when a patient is brought to the recovery room and continues until they are discharged from the hospital and start receiving follow-up care. In addition to the accelerated rehabilitation resulting from minimally invasive surgical procedures [77], the diversity in the research ecosystem has led to the development of new rehabilitation tools, enabling more efficient recovery of the patient’s health post-surgery.

#### 2.5.1. Technologies to Advance Manipulation and Mobility

The use of motors to aid human mobility dates back many years, although powered devices are often still cumbersome and expensive, particularly those used in hospital-based rehabilitation. For instance, continuous passive motion and robotic equipment are coupled with electromagnetic sensor technology to measure upper- and lower-limb kinematics during repair. However, rehabilitation following surgical procedures often occurs at the patient’s home. As a result, the development of relatively affordable, user-friendly, and generally accessible inertial sensors, cell phones, software applications, and commercial gaming hardware has become popular. Moreover, it may be crucial to monitor patients remotely [78].

#### 2.5.2. Hospital Rehabilitation

Continuous passive motion (CPM) is a treatment in which a machine moves a joint without the patient’s participation. After surgery, regaining normal joint mobility may be a substantial task [79,80]. Additionally, joint stiffness may impede recovery and cause discomfort. CPM is provided using a motorized device that commonly bends the joint a predetermined number of degrees, and the physical therapist controls the amount of movement and the pace.

CPM can be coupled with electromagnetic sensors. Using a transmitter, a six-degrees-of-freedom measurement device concurrently records the position and orientation of several electromagnetic sensors connected to body segments [81]. The ETS may also be used to assess the efficacy of rehabilitation programs by measuring kinematic changes in patients over time. For example, a more clinically friendly system for measuring knee kinematics with only two electromagnetic sensors (on the thigh and shank) has recently been designed to examine knee kinematics during pivot-shifting, delivering information on six-degrees-of-freedom knee kinematics at a high sample rate. Technology has also been used to quantify rotational laxity for clinical follow-up and research following ACL repair [78].

**Figure 6:**
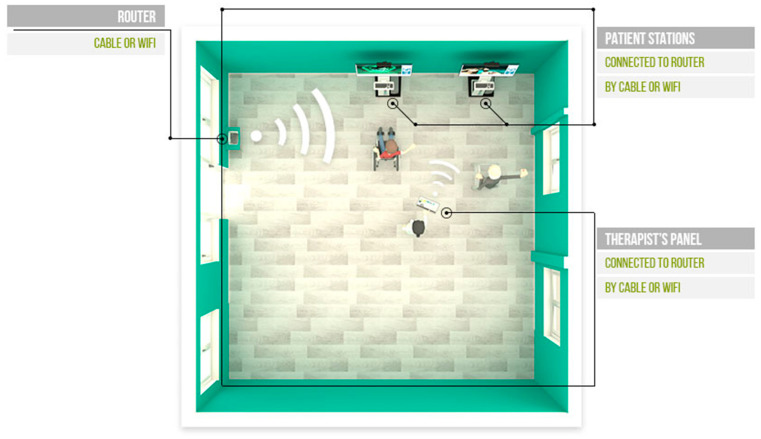
Setup for the VAST rehabilitation system. Router, Patient Stations, and Therapist panels are shown in a physical therapy room. The therapist panel and the various patient stations are equipped for seamless, direct communication, enabling the therapist to adjust gamification parameters dynamically in real time. This interaction provides a tailored experience for each patient, enabling personalized adjustments that enhance engagement and therapeutic outcomes. Source: VAST.REHAB.

**Figure 7:**
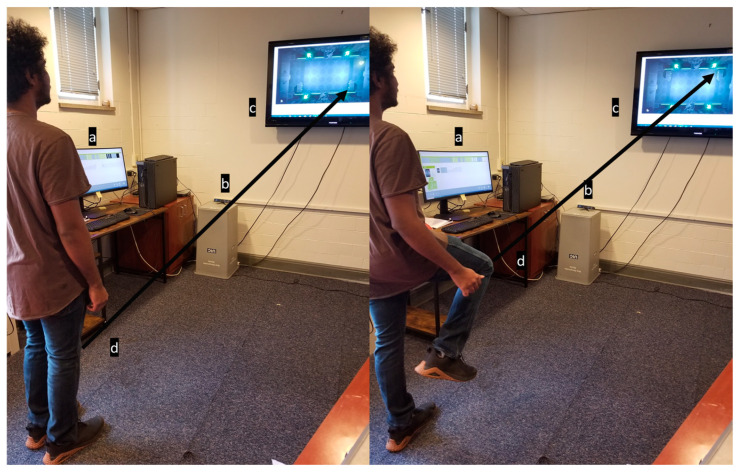
Example of a VR system utilizing game-based exercises: Subject controlling a Pong paddle on the screen by raising and lowering their leg during gameplay. The VR system utilizes game-based exercises as part of the Serious Games for Therapy model to enhance patient engagement and satisfaction [82]. The system components consist of (a) computer monitor, (b) Camera, (c) Interactive display screen and (d) Knee marker.

**Figure 8:**
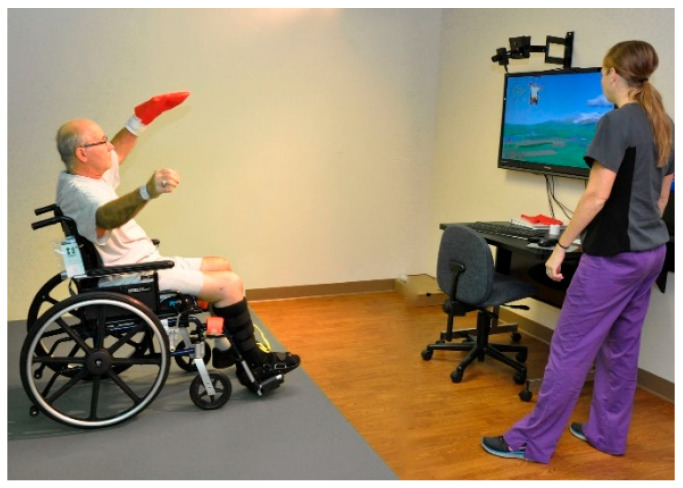
This VR system can track upper- and lower-body movements and features pre-built functions similar to physical therapy exercises. The system enables a physical therapist to select patient-specific motions and games that best match the patient’s needs and goals [83]. Source: GestureTek’s IREX Immersive Rehabilitation Exercise Systems.

Some patients’ complexions are complex, especially for the therapist, who must support the patient’s body weight throughout recovery. Robotic supporting devices have been developed to reduce the physical demands on therapists. EKSO Ionics (San Rafael, CA, USA) has developed the first FDA-cleared exoskeleton designed to assist patients with significant lower-extremity paralysis relearning to stand and walk during rehabilitation. The non-tethered exoskeleton cannot be used by those who cannot finish standard mobility therapy without assistance. ReWalk Robotics (Marlborough, MA, USA) created a battery-powered system with a lightweight, wearable exoskeleton with motors at the hip and knee joints. The ReWalker moves subtly by changing the patient’s center of gravity. The device detects a forward tilt of the upper torso. It begins with natural functional body-shifting and generates a sequence of steps that mimics the natural functional gait of the legs. This transformation is showcased in Figure 6, Figure 7 and Figure 8, illustrating advanced rehabilitation devices.

#### 2.5.3. Home-Based Rehabilitation: The Application of Virtual Reality in Post-Surgical Physical Rehabilitation

Physical therapy is a staple of rehabilitation following an injury. This is true for both non-surgical and surgical injuries and rehabilitation. However, access to therapy can be limited for many due to the limited availability of appointments and the time constraints of physical therapists. Additionally, while traditional physical therapy (PT) is widely recognized as the gold standard in rehabilitation, there is still room to improve outcomes and utilize modern technology to enhance the foundation on which PT is built today. One area that has seen increased usage as an adjunct to physical therapy (PT) is virtual reality (VR)-assisted therapy. For instance, some rehabilitation systems enable patients to communicate remotely with their therapist, as shown in Figure 6. VR has several potential benefits that make it an ideal solution for addressing the current gap in access to care. First, an off-the-shelf system can be deployed in many settings and is much closer to patients than traditional physical therapy (PT). Additionally, it offers far more specialized and individualized therapy than a conventional physical therapy protocol, as the system provides feedback on progress and current deficiencies throughout therapy. Virtual reality (VR), particularly in the context of the Serious Games for Therapy model, has also demonstrated significant benefits in terms of patient engagement and satisfaction [82]. Figure 7 shows an example of a VR system with game-based exercises, highlighting the exciting potential of VR in the future of rehabilitation.

Patients’ functional outcomes improve above those achieved with traditional physical therapy (PT) alone, indicating that VR is a viable adjunct to conventional PT and offers added benefits.

Patient outcomes in the post-surgical phase are now driving the development of integrated orthopedic platforms. The feedback of medical professionals, researchers, and students is crucial in this process. Patient feedback and data analytics help refine the entire process, from preoperative planning to recovery, ensuring that the platforms we develop are genuinely patient-centered and effective.

For any evaluation of VR, a decision must be made about its purpose and cost. An off-the-shelf VR system with options for adding accessories and sensing is best suited for any new developments. For example, using a headset could cause patients to become disoriented when performing lower-body exercises. So, a system without any headset was evaluated. This VR system can track lower-body movements and features pre-built functions similar to those used in physical therapy. The selected system enables a physical therapist to choose patient-specific exercises and games that best align with the patient’s current needs and goals. Patients will be evaluated using functional outcome measures, such as time to get up and go and the 10 m walk test, to assess improvement throughout therapy.

To assess the usefulness of virtual reality (VR), patients will undergo either traditional physical therapy (PT) or VR-based PT, and their functional outcomes will be evaluated before and after treatment. The therapy will vary depending on the patient’s injury or surgery, so no one protocol will be used alone, as shown in Figure 8.

### 2.6. Orthopedic Platform and Patient Feedback

It is estimated that 45,000 orthopedic surgeons are actively working in the United States. The Association of American Medical Colleges (AAMC) projects a shortage of 122,000 physicians by 2032. Most surgeons retire at 65, but a few retire much later.

In the future, in-person visits will increasingly transition into virtual visits, and additional doctors will be needed to handle the increased volume of calls. Regardless of patient care needs and circumstances, medical technology continues to advance at a rapid pace; therefore, healthcare institutions must adapt to these changes. Digital platforms are being developed to address the need to connect patients and doctors, as well as, potentially, orthopedic manufacturers and rehabilitation centers, thereby defining a new path to medical care.

**Figure 9:**
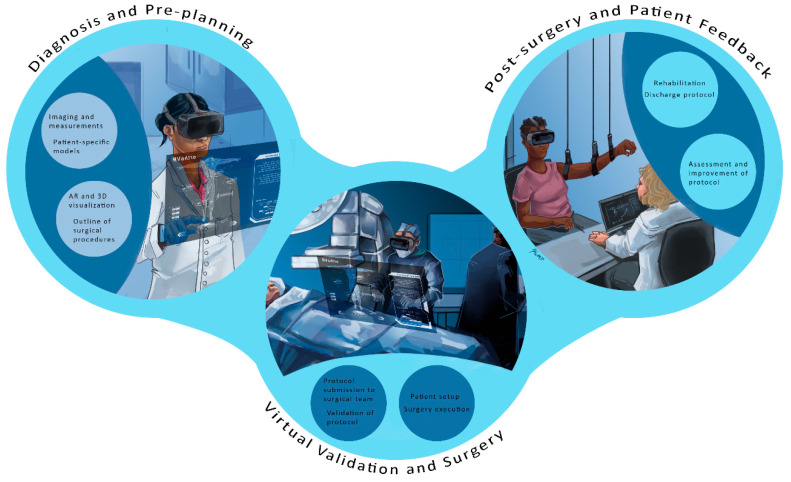
The orthopedic surgical future platform: Future platforms will integrate virtual visits, pre-planning simulations, and custom 3D printing to enhance precision and outcomes. The future of orthopedic surgery is set to transform with innovative platforms that integrate virtual consultations, advanced pre-planning simulations, and custom 3D printing. These technologies will enhance surgical precision and improve patient outcomes. Virtual visits will enable more accurate assessments, while pre-planning simulations will ensure that every detail is considered before surgery. Custom 3D printing offers precise models tailored to each patient’s unique anatomy, redefining the surgical landscape for safer and more effective procedures.

The orthopedics platform is defined by the current trends taking shape in several hospitals nationwide. Future orthopedic platforms will integrate virtual visits, pre-planning simulations, and custom 3D printing for implants. These innovations aim to enhance surgical precision and patient outcomes, as illustrated in Figure 9, which shows the concept diagram of the orthopedic platform. As emphasized in the early part of this paper, the structure is divided into several phases that include the following:

(a) Virtual orthopedic visits, where patients can get the care they need in the comfort of their own homes. Various healthcare providers are developing telehealth services to facilitate remote medical visits. Patients can shop around depending on their needs and schedule visits as needed.

(b) Advanced technology and surgical pre-planning: A combination of AR, VR, and mixed reality tools will be utilized in a simulation center environment, where 3D anatomical images are specifically created for each patient. Hence, a pre-planning surgical protocol can be developed. Doctors and their surgical medical team will gain firsthand experience without entering a live operating room. Monitoring devices can be added to enhance the surgical experience when needed.

(c) Patient-specific 3D-printed implants. The planning phase enables the selection and virtual fitting of implants, allowing surgeons to custom-fit patients with the assistance of their research staff. Implants are readily available if manufactured locally at the hospital, provided they meet FDA requirements and adhere to sterilization procedures. Major orthopedic companies can work closely with hospitals to meet quality care, implant printing, and quality control requirements.

(d) Surgical protocol plan and validation. The pre-planning enables the surgeon to generate and validate a surgical plan, which is then submitted to all relevant surgical staff, including anesthesiologists, nurses, and physician assistants (PAs). Most of the surgical team will be able to review and prepare for the surgical phase.

(e) Surgical phase. Future operating rooms will be transformed to accommodate technologies such as AR, VR, robotics, monitoring devices, imaging capabilities, and a network for potential virtual assistance. The surgical planning phase ensures that the setup and readiness are as outlined in the surgical protocol. In this new operating theatre design, procedures should be quick and easy to minimize time and optimize patient care outcomes.

(f) Rehabilitation and feedback. Patient rehab is crucial for a quick return to normal activities, and a combination of assistive devices and monitoring will play a significant role in data collection and feedback. Computerized rehabilitation protocols can be combined with virtual reality (VR) to provide patients ample exercise time at rehabilitation centers and home. Different surgical procedures can be evaluated based on patient feedback, which may feed into potential changes in the pre-surgical phase. The objective is to benefit the patient, allowing the surgeon, as is the case with TKA and THA, to spend less time in surgery, with faster patient recovery, while achieving a greater range of motion and longer-lasting implants.

## 3. A Look into the Future

Orthopedic surgery is shifting toward less invasive and more precise procedures, increasing outpatient and overnight procedures. In addition, the current and future development of innovative technologies will make surgery more selective and the orthopedic surgeon more focused on caring for injuries and diseases.

Collaboration between clinical and technological leaders will be essential for the future of orthopedic research and applications. As discussed, the interaction between the two entities resolves complex patient health concerns by designing adaptive models to comprehend the condition and individualized treatment and rehabilitation methods. Currently, orthopedic residency programs are developing a unique set of orthopedic surgeons who increasingly rely on technology.

Digital learning refers to the educational use of electronic media and digital technologies. It encompasses computer-based, internet-based, web-based, online, and virtual education through a Virtual Learning Environment (VLE), which comprises online tools, databases, and control resources that work in conjunction to support teaching and learning. It has been demonstrated that digital learning is more effective, less expensive, and more satisfying for students than traditional approaches [84]. However, digital learning cannot replace direct consultant supervision at surgical trainees’ workplaces, and blended learning has proven to be the most effective method.

The impact of nanobiomaterials is a work in progress. The clinical benefits of nanobiomaterials science require a deep understanding of the cellular and molecular basis governing the function of nanostructures and cells. While this topic is receiving considerable attention, challenges at all levels are still being investigated to develop an optimized structure with properties that mimic those of natural bone or cartilage and respond to load and stress without premature failure.

The application of nanotechnology combined with bioprinting is a new frontier in orthopedic research. The future of these technologies holds immense potential to enhance current orthopedic biomaterials and facilitate the development of innovative tissue engineering scaffolds and tissue regeneration solutions for muscles, tendons, bones, and biodegradable implants.

- Towards more automation:

The operating room is changing. The continuous development of augmented reality and artificial intelligence will enable intelligent, automated workflows and more effective robotic operations with real-time feedback.

Automation will enable the collection of patient information, the conversion of 2D images into 3D models, and the easy sharing of data between surgeons and biomedical engineers, instilling confidence throughout the entire surgical team. Proper planning before surgery also enables intelligent sizing, resulting in reduced inventory needs during surgery and a more successful outcome. With automated, patient-specific, 3D-printed implants, surgical instruments will be tailored to the individual patient.

- Description of implant design and Finite Element Analysis (FEA)

Implant design in orthopedics focuses on creating biomedical devices that can effectively replace or support damaged tissues, enhancing patient outcomes. The design process involves understanding the anatomical and biomechanical requirements specific to the injury or condition being treated. Key factors include material selection, geometry, and surface properties, which are crucial for ensuring the implant’s biocompatibility, mechanical strength, and longevity.

Finite Element Analysis (FEA) plays a crucial role in optimizing implant design. This computational technique allows for the simulation and analysis of implants’ behavior under various physiological loads and conditions. Using FEA, designers can identify stress distributions, potential points of failure, and areas for improvement in the implant’s structure.

- The optimization process involves several steps:

1. Model Creation: A detailed CAD model of the implant is developed, incorporating anatomical features and the desired specifications for the implant’s functionality.

2. Mesh Generation: The CAD model is discretized into smaller elements, creating a finite element mesh that enables detailed stress and strain analysis across the entire implant.

3. Boundary Conditions and Loads: Realistic boundary conditions, including forces generated by muscle activity, weight-bearing, and other physiological factors, are applied to simulate the environment where the implant will operate.

4. Simulation and Analysis: Using FEA software, various loading scenarios are simulated to determine how the implant performs under different conditions. This analysis helps identify areas of high stress and potential failure.

5. Optimization Iteration: Based on the FEA results, the implant design may be iteratively adjusted to reduce stress concentrations, enhance stability, and improve overall performance. This can involve changing geometrical features, selecting different materials, or modifying the surface texture.

6. Validation: Once optimization is complete, the final design must be validated through physical testing, such as mechanical testing and biocompatibility assays, to ensure it meets the necessary standards for safety and efficacy.

By integrating advanced computational techniques, such as FEA, the design of orthopedic implants becomes more precise and tailored to individual patient needs, ultimately leading to improved surgical outcomes and an enhanced quality of life for patients.

AI technologies will give surgeons abilities they have never possessed before. Automation also facilitates the smooth flow of communication and storing all data in a readily accessible and organized format while managing surgery by keeping track of all inventories and ensuring that all components arrive at the hospital or ASC on time. Inventory is another factor that can occasionally cause surgical delays.

**Figure 10:**
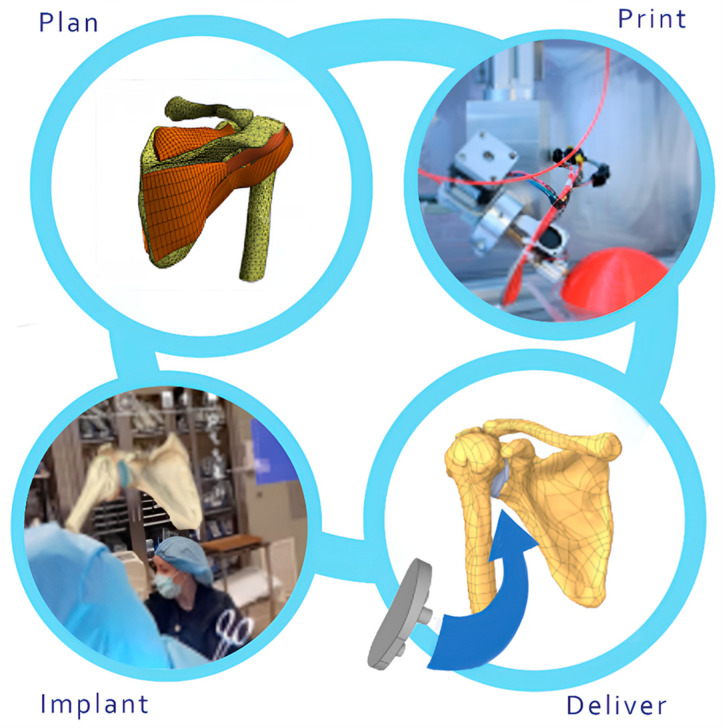
Processing patient-specific implants: Workflow from imaging to 3D printing and surgical application. Automation and AI transform surgery with real-time simulations and personalized implants.

Automation and AI will redefine the surgical landscape, enabling the development of patient-specific implants and facilitating real-time simulations. This vision is captured in Figure 10, which illustrates the flowchart of the process from patient-specific imaging to 3D printing and surgical application.

Thanks to patient-specific instruments and implants, improved surgical outcomes and quicker recoveries are now possible during the treatment or surgical phase. Additionally, augmented reality will enhance the visualization and understanding of the condition, improving the surgeon’s precision and reducing errors.

The post-treatment phase is also changing, thanks to user-friendly applications accessible via smartphones and wearable devices. These allow the medical team to monitor the patient’s real-time health condition and adjust the treatment.

**Challenges, Risks, Costs, and Implementation**

Technical Limitations: Many VR systems may not accurately replicate real-world scenarios, potentially creating a gap in training effectiveness. Furthermore, issues with system compatibility and integration with existing healthcare technologies can hinder usability.

User Acceptance: Patients may be reluctant to engage with new technologies, particularly older adults who may not be comfortable with virtual reality (VR) or telehealth systems. This could impact participation rates and overall satisfaction with therapy.

Data Security and Privacy: As more patient data are shared on digital platforms; the risk of data breaches is heightened. Ensuring the privacy and security of patient information is critical to maintaining trust.

Cost of Implementation: While the long-term benefits may be significant, the initial costs for advanced systems and training can be prohibitive for some healthcare institutions. These costs include software licensing, hardware purchases, and ongoing maintenance expenses.

Regulatory Compliance: Meeting FDA regulations for new devices and therapies can be lengthy and complex, potentially delaying innovative technology adoption.

Dependence on Technology: Over-reliance on virtual platforms may lead to a lack of hands-on training for medical professionals, potentially reducing the quality of care in cases where in-person evaluations are necessary.

Initial Investment: The upfront costs of purchasing VR, AR, or mixed reality systems can be high. These costs include hardware (headsets and sensors), software (licenses and subscriptions), and necessary accessories.

Training Expenses: Staff will require training to utilize new technologies effectively. This can involve initial training sessions and ongoing education as systems are updated or expanded.

Maintenance and Upgrades: Regular software updates and hardware maintenance will incur ongoing costs. Institutions must allocate funds for these expenditures to ensure their systems remain functional and secure.

Telehealth Infrastructure: Implementing virtual visits requires an investment in the necessary infrastructure, including reliable internet connections, digital platforms, and security measures to protect patient data.

Pilot Programs: Initiating small pilot programs can help gauge the effectiveness of new technologies before implementing them on a full scale. This allows for adjustments based on feedback and can help overcome resistance from staff and patients.

Stakeholder Engagement: It is crucial to involve all stakeholders, including healthcare professionals, patients, and IT teams, in the planning and implementation process. Their input can inform design choices and increase buy-in for the technology.

Integration with Existing Systems: Ensuring that new platforms can integrate seamlessly with current electronic health records and other healthcare systems is essential for providing a cohesive experience for staff and patients.

Feedback Mechanisms: Establishing robust systems to gather patient and provider feedback can help continuously refine the technology and its applications based on real-world experiences.

Regulatory Planning: Healthcare institutions should plan for regulatory approval timelines early to avoid implementation interruptions due to compliance delays.

Healthcare facilities can effectively leverage advanced technologies to enhance patient care in orthopedics and rehabilitation by thoroughly addressing these challenges and costs during the planning and implementation stages.

## 4. Discussion

The future of orthopedic research and basic science will be significantly impacted by the rapid increase in the use of patient-specific printing techniques for implants, materials, imaging, and sensory feedback tools, such as navigation, tracking mobility, and sensor support for diagnosis and balance, as seen in total knee arthroplasty (TKA). This technology will help us better understand how complex surgeries are performed and render primary Total hip arthroplasty or Total knee arthroplasty routine. Artificial intelligence works best when a highly advanced orthopedic platform is fully operational. Training, education, and performance optimization of skills and procedures will benefit from AI and other computer-generated algorithms. As large databases are created around various patient pathologies, diagnosis and surgical planning improvements will lead to a dramatic shift toward automation and technological integration that has never been seen before.

The role of biomechanics has expanded beyond testing and experimentation in the last decade. CT, segmentation, and 3D modeling have created a new paradigm in simulation, extensive use of FEA, and patient-specific design of implants. While virtual testing is becoming the norm, it has bridged into surgical pre-planning and augmented reality. Surgeons will be able to interact with a natural environment and request immediate answers from AR simulations and FEA. As new products are introduced, feedback will be sought on the engineering of bone–implant interfaces and how new implant materials affect human joint function. This interactive simulation, which allows for real-time surgery, is made possible through real-time simulation and analysis.

These new in silico ideas will add a new dimension to residents’ training and knowledge, enabling future surgeons to collaborate on complex surgical procedures. In parallel, the orthopedic industry, medical device manufacturers, implant suppliers, and surgical tool developers will integrate virtual laboratory AR and VR into their existing platforms to optimize their design.

COVID-19 has required medical professionals to work remotely. It has, in turn, created ideas, tools, and resources that have compelled hospitals to collaborate with the private sector to develop new platforms and opportunities that were previously unavailable. Technology is constantly evolving and will continue to be in high demand. However, maintaining course and training our surgeons to stay abreast of modern technology integration for remote and virtual patient care is crucial to elevating surgical outcomes and patient satisfaction.

In contrast to the revolution of novel technologies and devices derived from basic science in orthopedics, clinical life’s evolution appears to be slower. A challenge of clinical research in orthopedics is the large variety of patients with increasing treatment options offered by basic science. Therefore, international, industry-independent, randomized controlled trials must prove the clinical relevance of new and existing devices and therapies on the market that are derived from basic science.
